# Diet composition and diversity does not explain fewer, smaller urban nestlings

**DOI:** 10.1371/journal.pone.0264381

**Published:** 2022-03-01

**Authors:** Erin E. Grabarczyk, Sharon A. Gill, Maarten J. Vonhof, Magdy S. Alabady, Zengyan Wang, Jason M. Schmidt

**Affiliations:** 1 Department of Biological Sciences, Western Michigan University, Kalamazoo, MI, United States of America; 2 Institute of the Environment and Sustainability, Western Michigan University, Kalamazoo, MI, United States of America; 3 Department of Plant Biology, University of Georgia, Athens, GA, United States of America; 4 Entomology Department, University of Georgia, Tifton, GA, United States of America; City University of New York, UNITED STATES

## Abstract

The reproductive success of animals breeding in cities is often lower compared to counterparts that inhabit rural, suburban, and peri-urban areas. Urban dwelling may be especially costly for offspring development and survival. Diet composition and diversity may underlie factors that lead to lower fitness, particularly if prey abundance and quality decline in modified environments. Moreover, breeding success may change over the course of a season, an effect that may be augmented in urban areas. In this study, we tested the hypothesis that habitat and date affected nestling house wren (*Troglodytes aedon*) body condition and survival, and examined whether diet explained differences in nestling success. We monitored urban and rural populations of house wrens breeding in nest boxes, and tested whether clutch size, nestling survivorship, and nestling body condition varied by habitat or by date, and then characterized the diet of a subset of nestlings with DNA metabarcoding of fecal samples. Urbanization had clear impacts on house wren nestling fitness: urban broods contained fewer, smaller nestlings. Early nestling survival decreased as the breeding season progressed, and this effect was more pronounced in the urban population. However, the diets of urban and rural nestlings were similar and did not explain differences in body condition. Instead, across populations, diet changed with date, becoming less diverse, with fewer Lepidoptera and more Orthoptera. Regardless of habitat, adult house wrens provide nestlings with similar types of foods, but other factors, such as quantity or quality of prey delivered, may lead to fitness disparities between urban and rural nestlings.

## Introduction

Across taxa, human-generated landscape transformation has resulted in significant declines in biodiversity and species abundance (arthropods: [1–3]; mammals, reptiles, plants, amphibians: [4]). Novel urban environments differ from natural habitat in which animals have evolved, altering the conditions for selection. Correlative evidence suggests that the reproductive success of many animals that breed in cities is often lower compared to counterparts that inhabit less modified environments, such as rural, suburban, and peri-urban areas [5, 6]. In urban areas, observed fitness differences may result from selective filtering of individuals within populations based on their ability to adjust to new environmental conditions [7]. Alternatively, poor fitness outcomes may instead be due to lack of or lower quality resources in cities, such as space, food, or mates [8, 9]. Although factors such as pollution (e.g., noise, light, or chemical), habitat loss and change, and disease may contribute to settlement or fitness declines, identifying the mechanisms that underlie maladaptive responses is critical for the conservation of biodiversity [10, 11].

The consequences of urban dwelling may be especially costly for the development and survival of offspring. For birds, factors that reduce nestling health in urban ecosystems are diverse, for example, ranging from increased stress [12, 13] and cholesterol [14] to decreased body condition [15, 16]. In terms of health, the composition and diversity of arthropods in nestling diets may underlie patterns of poor physical condition and reduced fledging success. Arthropod prey items vary in caloric or nutritional value as well as palatability [17, 18], and for many species, nestling survival and body condition is linked to the availability of high-quality, preferred prey items [16, 19]. Urbanization significantly decreases arthropod populations [1] and can result in reduced abundance of high-quality prey [20]. Moreover, seasonal changes in prey and avian diet patterns may be related to local habitat composition [21]. In urban areas, this may negatively impact nestling fitness, if such areas experience a larger overall drop in the availability of high-quality prey as the breeding season progresses.

Traditional diet sampling techniques oftentimes fail to capture how the diets of individuals change over time, or alternatively, cannot make a direct link between diet and fitness outcomes. For example, analysis of stomach contents (via neck ligatures or dissection) allows for prey identification, but due to the nature of sampling, typically does not permit sampling of the same individuals over multiple time points [22–24]. Tracking diet based on observation of adult provisioning behavior is non-invasive, but may fail to reveal the diets of individuals, and instead summarizes diet for an entire brood [25, 26]. DNA fecal metabarcoding is a non-invasive technique that also allows for monitoring the diet composition of individuals at multiple time points [27, 28]. This approach enables a direct link between fitness traits of individuals, such as body condition, survival, and growth, to consumed prey items detected from gut content samples. Furthermore, diet composition can be quantified based on presence and absence of prey items as well as the relative read abundance, a proportional summary of counts, which in some instances, can be used to approximate the amount of prey in an individual’s diet [29].

Here, we tested the hypothesis that nestling house wren (*Troglodytes aedon*) condition and survival differs between urban and rural habitats, and examined whether diet explained differences in nestling success. We first established whether breeding habitat and time of year affected reproductive outcomes between an urban and a rural population of house wrens breeding in southwest Michigan. For two years, we monitored house wrens breeding in nest boxes and tested whether clutch size, early nestling survivorship, and nestling body condition varied by habitat and date. Next, we tested whether nestling diet diversity and presence of common prey items contributed to the predicted differences in nestling fitness based on habitat and date. For a subset of nestlings, we used DNA metabarcoding to characterize the diets of nestlings by analyzing fecal samples collected from younger (5–7 days post hatch) and older (8–12 days post hatch) nestlings. We collected fecal samples at two time points, because the amount of prey provisioned to house wren nestlings changes as they age, and the quality of prey early on affects later nestling growth [30]. We used day 7–8 post-hatch as our cut-off between younger and older nestlings: at less than seven days post hatch, nestling house wren growth is exponential, whereas after eight days, growth begins to asymptote [31, 32]. We predicted that nestlings in urban habitat to be in poorer condition than their rural counterparts, which may be due to the composition and diversity of their diets.

## Materials and methods

### Study species and sites

House wrens are a cavity-nesting, insectivorous passerine, that are distributed across a large latitudinal range (Tierra del Fuego to British Columbia). Clutch sizes vary with latitude; tropical subspecies produce fewer, smaller clutches compared to temperate subspecies [33]. Across their breeding range, seasonal prey availability, measured indirectly as actual evapotranspiration, does not predict clutch size [33], however presence of preferred prey and diet diversity may underlie breeding success. Like many other songbirds, Lepidoptera (caterpillars and moths) are an important food source for nestlings early in the breeding season [34–36]. However, house wrens in southwest Michigan initiate clutches over a three-month period (late April to late July), during which time insect abundance may vary. In addition to Lepidoptera, observation of house wren provisioning behavior suggests that adults commonly provide their nestlings with a variety of arthropods including crickets (Orthoptera) and spiders (Araneae) [37, 38]. Therefore, adults may switch provisioning to alternative prey as the season progresses and Lepidoptera are less abundant [39, 40], which could have downstream effects on nestling success.

From 2016–2017, we monitored two populations of house wrens breeding in nest boxes (n = 48 rural, 42 urban) in Kalamazoo County, Michigan, USA (S1 Fig). Our rural site, Chipman Preserve (42.307°N, 85.461°W, n = 48 boxes), is a single 93 ha natural area, surrounded by low-density housing, agricultural fields, a commercial green house, and adjacent to a single-lane county road on one edge of the preserve. The urban population of house wrens was split between two adjacent sites, Asylum Lake Preserve (42.260°N, 85.637°W; 110 ha, n = 24 nest boxes) and natural areas surrounding Western Michigan University’s Parkview campus (42.253°N, 85.634°W; 17 ha, n = 18 nest boxes), which are located across a two-lane road from one another (S1 Fig). All nest boxes at the urban sites were within 400 m of a local road and within 730 m of a major four-lane highway. Compared to both urban locations (Asylum Lake and Parkview), Chipman Preserve has on average lower levels of anthropogenic noise pollution [41], artificial light pollution [42], as well as lower percent impervious surface surrounding the preserve (S1 Fig). Within sites, nest boxes were arranged in open habitat near forest edge as 6-box groups separated by 45–50 m at a 60° angle. Each group of nest boxes was separated by at least 150 m [41].

### Monitoring nest box occupancy and nestling fitness

Nest boxes were checked every three days to determine occupancy and to monitor house wren breeding progression. During nest checks, we recorded date of first occupancy by adults, clutch initiation, number of eggs laid, hatch dates, number of nestlings to hatch, monitored nestling growth, and noted predation events. All nestlings that were not included in the diet analysis (see below) were banded on days 8–13 post hatch. We measured nestling mass (g) with a Pesola Micro Spring Scale (0.25 g accuracy) and tarsus length (mm) with a dial caliper (SPI Polymid Dial Caliper; 0.1 mm accuracy). To quantify nestling body condition, we extracted the residuals from a regression of mass and tarsus, and corrected values by the age of nestlings on the day of banding ([43]; mean ± SD nestling age on day of banding: 9.9 d ± 1.0). Based on this index, we considered nestlings with a positive residual value to be in better condition than nestlings with a negative residual value.

### Fecal sample collection

In 2017, we measured body size and collected fecal samples from a subset of nestlings at two time points prior to fledging (n = 129 nestlings from 26 clutches). The first collection took place early in the nestling period, at 5–7 days post hatch (hereafter, young nestlings). At this age, the eyes of nestlings have opened, but primary feathers have not yet started to develop. For the subset of nestlings included in the diet analysis, young nestlings were banded with a numbered USGS silver aluminum band. The same individuals were measured a second time at 8–12 days post hatch (hereafter, older nestlings). At this age, nestlings have developed body tract feathers as well as primary feathers. At both sample points, we measured nestling mass (g) and tarsus (mm). To collect fecal samples, nestlings were carefully removed from the nest box and placed individually into autoclaved brown paper holding bags. To reduce environmental contamination, all nestlings were handled with nitrile gloves, which were sterilized with a 70% alcohol pad between individuals. Nestlings often defecated almost immediately upon handling; therefore, the majority of fecal samples were collected when nestlings were first removed from the nest box and prior to being placed in a holding bag. If nestlings did not defecate immediately, they were kept in the holding bag for 5–10 min, during which time brood-mates were processed. If nestlings defecated in holding bags, then fecal samples were extracted with forceps that were sterilized between uses. All fecal samples were preserved in autoclaved 1.5 mL plastic vials that contained 500 microliters of RNAlater (ThermoFisher, USA). E. Grabarczyk conducted all nestling banding, body measurement, and fecal collection at both time points. During banding, each nestling was handled for less than 5-minutes to minimize stress and immediately returned to the nest following processing. Upon return to the lab, samples were stored in -20°C freezer until DNA extraction and processing.

### Molecular gut content analysis

Arthropod DNA was extracted from nestling fecal samples (including the fecal sac) with the PowerSoil kit (Qiagen, Hilden, Germany) following the manufacturer’s instructions. We selected the PowerSoil kit for extraction, as this approach has been identified as a universal extraction kit for a variety of sample types that produces high quality DNA [44]. For each batch of 96 samples, a blank extraction that did not contain fecal material was added as a negative control to account for background DNA. The same blank negative control from extractions was also used during PCR plate preparation. Each plate also contained a positive control with known arthropod DNA.

We used a two-step nested DNA metabarcoding approach to detect arthropod DNA in nestling fecal samples [45]. In the first round of PCR, DNA was amplified with the primer pair ZBJ-ArtF1c/ZBJ-ArtR2c [46], which targets a 157 base pair fragment of mitochondrial *Cytochrome Oxidase* subunit I (COI) gene and each sample was dual-tagged with a unique combination of eight forward and eight reverse tags [45]. Following this round, PCR products were cleaned with 1X AmpureBeads XP (Beckman Coulter Brea, CA USA). In the second round of PCR, a set of molecular tags were added to the amplified COI region as well as Illumina adapters. Each plate of fecal samples received forward and reverse tags that consisted of a unique eight nucleotide sequences and enabled demultiplexing of plates and individual identification of samples post-sequencing. PCR products were again cleaned and the concentration of amplicons was estimated (ng/ul) and visualized using the Qiaxcel Advanced System (Qiagen). The concentration of PCR products was standardized across samples and plates, and pooled. Pooled samples were submitted to the Georgia Genomics and Bioinformatics Core lab (GGBC-UGA) for sequencing on an Illumina MiSeq (Illumina, San Diego, CA, USA) with V2 chemistry and 300 cycles.

After quality trimming of the sequencing reads, we merged demultiplexed reads with PEAR [47] that had a minimum overlap of 50 bp and a minimum quality of 20. Following Schmidt et al. [48], we transformed reads that had at least 90% of the sequence with > Q30 quality into fasta files (FastX toolkit) and trimmed primer sequences with awk commands [49]. In USEARCH, we clustered Operational Taxonomic Units (hereafter; OTU’s) at a 3% similarity threshold [50, 51] and performed a *de novo* chimera removal. In MEGA, we translated OTUs [52], retaining those with an intact reading frame. We assigned taxonomy to the remaining OTUs by blasting them against the NCBI database and built OTU tables in USEARCH.

Based on OTU tables for reads of different taxa recovered from fecal samples, we identified prey items to the level of family and converted reads into diet metrics [29]. Consistent with Deagle et al. [29], we explored the data with both standardized counts of taxa in fecal samples (i.e., presence or absence) as well as rarefied relative read abundance (RRRA) to estimate diet diversity. To describe taxon occurrence, we set our threshold for presence to ten reads, such that any taxon in a sample that contained less than ten reads was adjusted to zero (i.e., absent) and greater than 10 reads was adjusted to 1 [53–55]. For our analysis of diet diversity, we took a conservative approach, using standardized rarified relative reads to control for variation in sequencing depth across samples. To determine the minimum threshold for standardized reads we used an iterative approach, eliminating reads that were present in low numbers, while still maintaining enough samples to test for patterns within our population (S2 Fig). This ensured we had sufficient sequencing depth to include all possible taxa nestling consumed, but that the threshold was not too stringent that it eliminated samples that may be biologically relevant. Based on RRRA, we visualized rank abundance of reads by taxa and calculated a rarified Shannon diversity index (H).

### Statistical analysis

#### Effects of date and habitat on nestling fitness

We used a generalized linear mixed effects model to test whether house wren clutch sizes varied between habitat and date. The model (glmer; family = Poisson, link = logit) included habitat (2 levels; urban and rural) and clutch initiation date (range: April 28—July 21) as fixed effects, and nest box identity nested within year (2 years; 2016–2017) as a random effect. We fit general linear mixed effects models to test whether nestling survivorship and body condition varied between habitat and date. As with clutch size, the model for survivorship also included habitat and clutch initiation date as fixed effects, and nest box identity nested within year as a random effect. For analysis of nestling body condition, we included habitat and hatch date (range: May 27 –August 7) as fixed effects, nest box identity nested within year as a random effect, and brood size as a covariate. Brood size may affect adult house wren provisioning rates and the amount of prey individual nestlings receive [30]. For all three models, we tested whether the interaction between habitat and date improved model fit by comparing Akaike information criteria corrected for small sample sizes (ΔAICc), but found that the interaction term did not improve fit for any of our models, therefore excluded this term from final models (S1 Table).

We tested whether diet metrics varied by habitat, date, and age, and whether the diet of nestlings predicted body condition. Our first diet model included a rarefied Shannon diversity index (H) as the response variable, with habitat, nestling age (2 levels; younger and older), and collection date as fixed effects, and nestling identity and nest box identity as random effects. Next, we considered whether common prey items in nestling diets varied based on habitat, nestling age, and date. For our models, we selected Lepidoptera and Orthoptera for analysis of common prey based on prior studies that have observed adult house wrens provisioning prey from these two orders at high rates across multiple study populations [37, 38, 56]. In addition, Lepidoptera and Orthoptera made up > 50% of the sequence data in our diet samples. Although spiders have also been identified as a common prey item during observation of provisioning, Araneae made up less than 10 percent of the DNA detected in our samples, and therefore was excluded from further analysis. To explore the effects of common prey present in nestling diets, we first fit generalized linear mixed models (family = Poisson, link = logit) that included rarified relative read abundance (RRRA) of Lepidopteran and Orthopteran as our response variables. Our fixed effects included habitat, nestling age, and date. Our random effects included nestling identity and nest box identity. We found that the data was over-dispersed, and as such, violated the assumptions of the model. Exploration of the data showed that the patterns of common prey detection by RRRA and taxa occurrence were similar (S3 Fig). Therefore, our final models that explored patterns of common prey items in nestling diets included the presence or absence of Lepidoptera and Orthoptera in nestling diets based on taxa occurrence. We fit two generalized linear mixed models (family = binomial) and included habitat, nestling age, and date as fixed effects. Nestling identity and nest box identity were included as random effects. Initial data exploration of the presence of Orthopterans in the diet by date suggested a non-linear pattern, therefore for this model we included a polynomial term for date. Our final general linear mixed model included body condition as the response variable and diet diversity (H), presence of Lepidoptera, and presence of Orthoptera as fixed effects. We included nestling identity and nest box identity as random effects. We centered and scaled continuous variables (date) prior to analysis, used histograms to assess normality, and examined residual plots to determine model adequacy. We report fixed effect coefficients and 95% confidence intervals and consider a fixed effect to influence nestling fitness if confidence intervals do not overlap with zero.

### Ethical treatment of animals

All experiments were approved by Western Michigan University’s Institutional Animal Care and Use Committee (IACUC No. 16-01-01).

## Results

**Fig 1:**
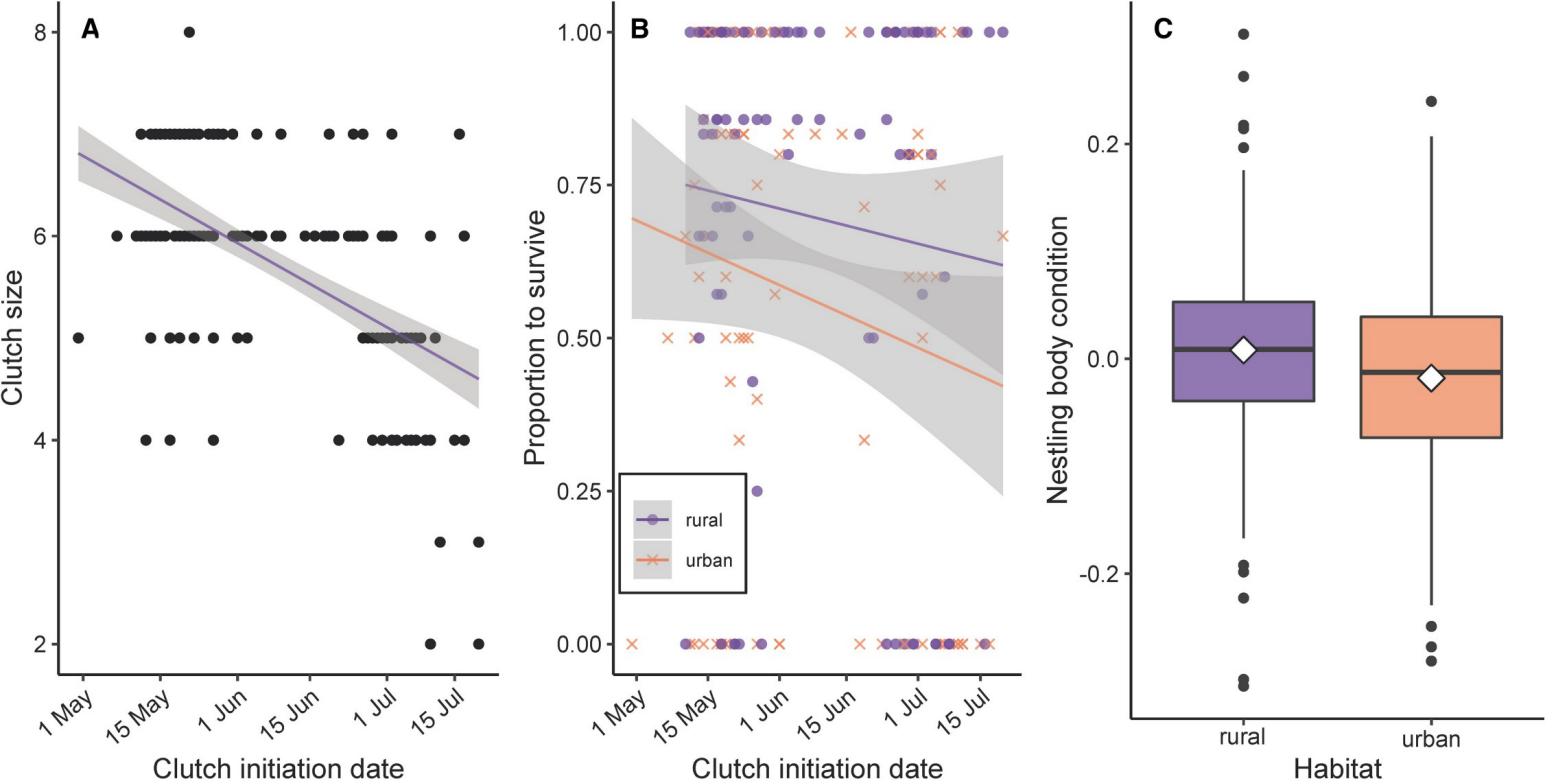
Reproductive success and nestling condition varied by date and habitat. Clutch sizes decreased with date, regardless of habitat (A). Nestling survival decreased with date, and this effect was more pronounced in the urban population (B). Regardless of hatch date, nestling condition was lower in urban habitat compared to rural (C; white diamonds with boxplot shows population mean).

Across two house wren breeding seasons, we confirmed 178 complete clutches (n = 89 urban, n = 89 rural clutches completed) and banded 678 nestlings (n = 284 urban, n = 363 rural). Regardless of habitat, clutch size decreased as the season progressed (Fig 1A and Table 1). Nestlings that hatched early in the season were more likely to survive from egg to day 10 post-hatch than those hatched in later clutches (Fig 1B and Table 1), with the decline in survivorship larger in the urban population compared to the rural population. Nestlings of rural clutches were in better body condition than urban nestlings. However, we found no evidence of a date effect on body condition (Fig 1C and Table 1). Of the completed clutches, 42 failed to fledge, which included 36 clutches that failed during incubation (n = 14 rural, n = 22 urban) and six that failed during the nestling stage (n = 3 rural, n = 3 urban).

**Table 1:** Fixed effect coefficients and 95% confidence intervals from general linear mixed effects models testing whether nestling success and condition varied according to habitat and date.

Model	Parameter	Estimate	Lower CI	Upper CI
Clutch size	Intercept	1.8	1.7	1.9
	Habitat (urban)	-0.08	-0.2	0.05
	**Clutch initiation date**	**-0.1**	**-0.17**	**-0.04**
Survivorship	Intercept	0.7	0.56	0.85
	**Habitat (urban)**	**-0.14**	**-0.25**	**-0.03**
	**Clutch initiation date**	**-0.06**	**-0.12**	**-0.003**
Body condition	Intercept	0.05	0.0004	0.1
	**Habitat (urban)**	**-0.03**	**-0.06**	**-0.01**
	Hatch date	-0.01	-0.02	0.002
	Brood size	-0.01	-0.01	0.002

### Diet in urban and rural populations

In 2017, we collected a total of 276 fecal samples from 129 nestlings (26 clutches; n = 6 urban and n = 20 rural). Post metabarcoding, rarefaction eliminated 102 samples from further consideration. Therefore, our total sample size for analysis of diet included 174 fecal samples from 113 individuals collected from 26 nest boxes; this sample included 11 younger and 18 older urban nestlings and 50 younger and 95 older rural nestlings.

**Fig 2:**
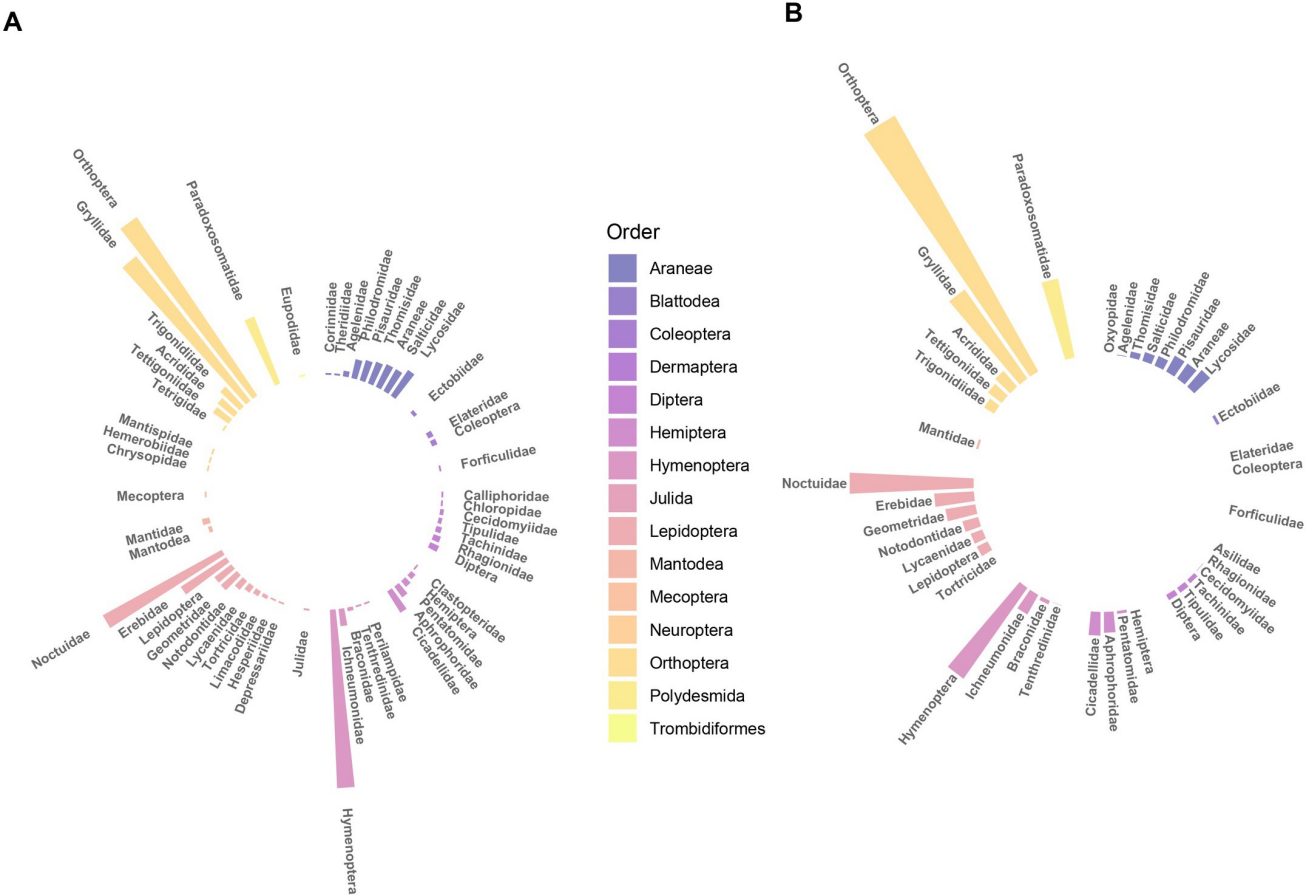
Circular barcharts representing rank abundance of (A) pooled frequency of occurrence (whether taxa are present or not based on >10 reads in a sample) for arthropod taxa in fecal samples, and (B) pooled rarefied reads for taxa observed in wren fecal samples.

Based on frequency of taxa counts, the most abundant prey items were Lepidoptera, followed by Orthoptera, Araneae, Polydesmida, and Hemiptera (Fig 2A). However, based on the distribution of total reads by taxa, the most abundant prey item was Orthoptera, followed by Lepidoptera, Polydesmida, Araneae, and Hemiptera (Fig 2B). Of the Orthoptera detected, crickets (Gryllidae) were the most common prey items detected in samples (n = 96 samples), Trigonidiidea (n = 15), Tettigoniidae (n = 11); S2 Table), followed by grasshoppers (Acrididae; n = 12 samples). Of the Lepidoptera detected, moths (Noctuidae (n = 71), Erebidae (n = 29), and Geometridae (n = 12)) were the most common prey detected in samples. The most common spiders among samples included Lycosidae (n = 17 samples) and Salticidae (n = 14). Leafhoppers were the most commonly detected Hemiptera (Cicadellidae (n = 14 samples). Out of all gut samples, 38 contained millipedes (Polydesmida: Paradoxosomatidae).

**Fig 3:**
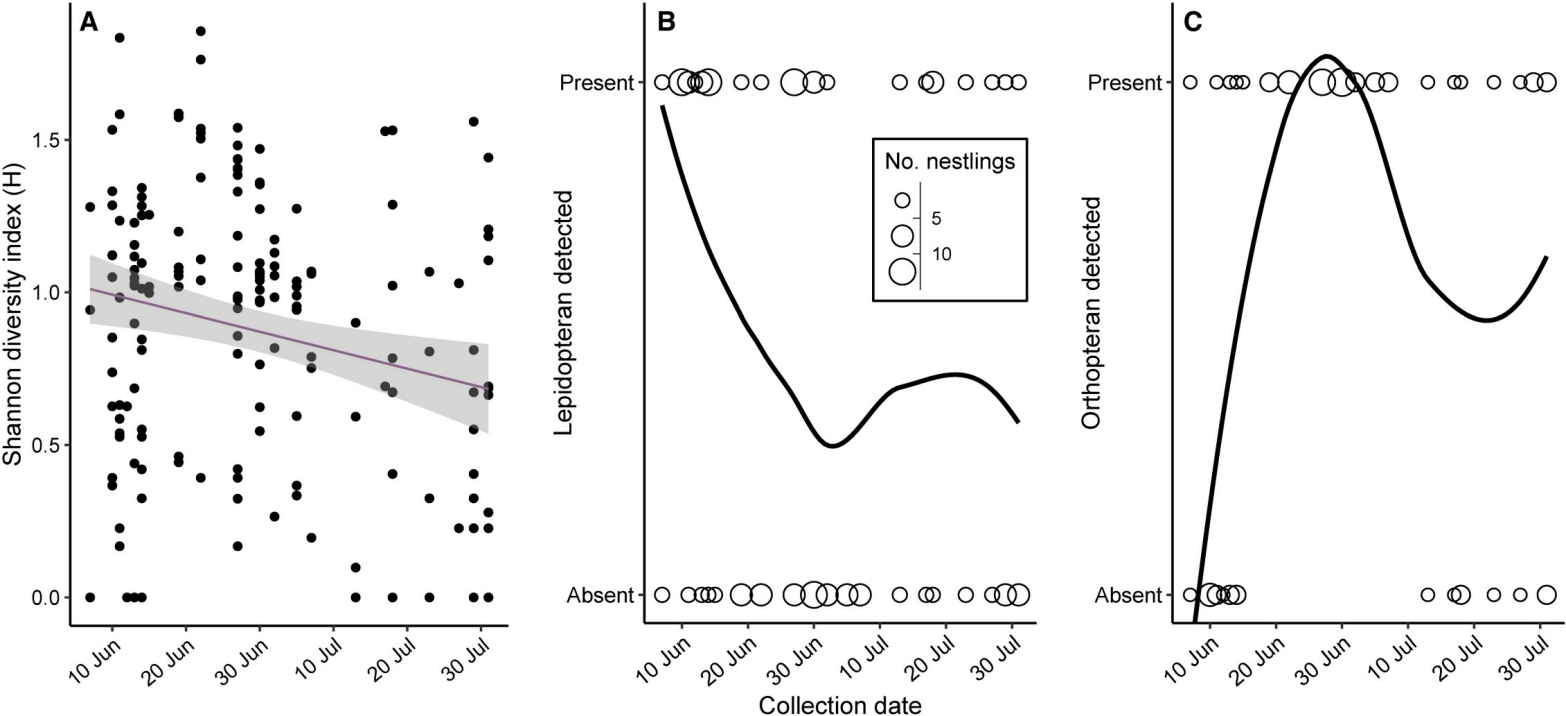
Nestling diet diversity and the presence of common prey items changed over time. The diversity of prey (H) in nestling diets decreased with date (A). Lepidopterans were present in nestling diets early (June), but declined over time (B), whereas Orthoptera were absent from nestling diets early, peaked around the same time that lepidopterans declined, then leveled out later (circle size is proportional to the number of individual nestlings with and without Lepidoptera (B) and Orthoptera (C) detected in their diet).

Diet diversity (Shannon diversity, H) and the presence of Lepidoptera detected in nestling fecal samples decreased with date (Fig 3A and 3B and Table 2). Lepidoptera present in nestling diets was high during early June, but started to decrease by mid to late June. Orthoptera present in gut samples increased at the same time that Lepidoptera declined (Fig 3B and 3C). Exploration of rarified relative read abundance showed the same seasonal pattern for Lepidoptera and Orthoptera (S3 Fig). Habitat and nestling age did not predict diet diversity or presence of common prey in nestling diets (Table 2). Finally, nestling body condition was not predicted by diet diversity or the presence of either Lepidoptera or Orthoptera in diets (Table 2).

**Table 2:** Fixed effect coefficients and 95% confidence intervals from general linear mixed effects models testing whether diet diversity measured as Shannon diversity index (H), presence of Lepidoptera, and presence of Orthoptera vary by habitat, nestling age, or date and whether body condition varies by diet metrics.

Model	Parameter	Estimate	Lower CI	Upper CI
SHDI (H)	Intercept	0.9	0.6	1
	Habitat (urban)	0.1	-0.1	0.3
	Nestling age (older)	-0.3	-0.2	0.01
	**Collection date**	**-0.1**	**-0.2**	**-0.02**
Lepidoptera	Intercept	1.4	0.2	3.0
	Habitat (urban)	-1.2	-2.8	0.2
	Nestling age (older)	-0.6	-1.3	0.1
	**Collection date**	**-0.03**	**-0.06**	**-0.004**
Orthoptera	Intercept	2.2	-0.7	4.4
	Habitat (urban)	1.9	-0.8	3.6
	Nestling age (older)	0.3	-0.7	1
	**Collection date**	**0.1**	**0.03**	**0.2**
	**Collection date^2**	**-0.01**	**-0.01**	**-0.004**
Body condition	Intercept	-0.3	-1.1	0.5
	SHDI	0.2	-0.5	0.9
	Lepidoptera	-0.4	-1.1	0.2
	Orthoptera	0.4	-0.4	1.2

## Discussion

Between study sites, urbanization had clear impacts on the survival and condition of house wren nestlings. Compared to their rural counterparts, urban broods contained fewer, smaller nestlings. In both populations, early nestling survival decreased as the season progressed; however, this effect was more pronounced in the urban population. Regardless of hatch date, average nestling body condition was lower in the urban population compared to the rural population, yet body condition of nestlings was not influenced by diet diversity or the specific presence of Lepidoptera or Orthoptera in the diet. Moreover, despite the habitat-related differences in overall success, nestling diet diversity, measured by the Shannon diversity index, did not differ according to habitat or nestling age. Instead, diet diversity decreased as the breeding season progressed, punctuated by a decline in Lepidoptera present in nestling diets, followed by a nearly simultaneous switch to Orthoptera.

Differences between urban and rural habitats may have a bottom-up effect on local biodiversity, from the type of vegetation present to the quality of arthropod prey consumed by insectivorous birds. Survivorship and body condition of house wren nestlings differed between habitats, and survivorship also varied according to the timing of nesting. In addition to smaller, late season clutches, nestling survival decreased as the season progressed, an effect that was more pronounced in the urban population. Diet diversity as well as the number of Lepidoptera present in nestling diets also declined over time. Particularly in temperate populations of house wrens, clutch size tends to decline with season [33]. However, food availability measured as actual evapotranspiration was not correlated with smaller clutches of house wrens across a latitudinal gradient [33]. Diet variation at the scale of latitude may be too large to predict clutch sizes, and other factors on a smaller scale, such as local habitat composition may better explain variation in clutch size. For example, on a local scale, many urban natural areas have fewer native trees that support preferred arthropods prey items for birds [57], which could lead to consumption of low-quality prey. Moreover, adult provisioning behavior may differ according to habitat composition, if prey densities vary. Adult pied flycatchers (*Ficedula hypoleuca*) breeding in urban habitat increased their search radius and search duration to find high-quality prey compared to a forest population [58]. Regardless of habitat, house wren nestling diet composition and diversity were similar. Therefore, urban nestlings may be provided with similar types of food, but the quality or quantity of food received by urban nestlings may be lower.

DNA metabarcoding of fecal samples revealed similar patterns of diet composition that have been previously described by observation of adult provisioning. Common prey items included Orthoptera, Lepidoptera, Hemiptera, Araneae (Fig 2; [37, 38]). In addition to common orders of insects, DNA metabarcoding allowed us to identify prey items to the level of family (S2 Table), which builds on our understanding of specific types of prey consumed by this species. Furthermore, analysis of nestling fecal samples via DNA metabarcoding showed similar patterns of prey items consumed by nestlings whether estimated with taxa occurrence or by rarified relative read abundance (RRRA). This suggests that prey items that were detected more often based on presence data also had the highest number of reads from molecular sequences. Therefore, either post-processing approach, taxa occurrence or RRRA, provided similar results in regards to dominant patterns of diet composition for characterizing nestlings diets.

Breeding birds may provide important ecosystem services in natural areas located near both urban and agricultural areas [59]. House wren nestlings consumed prey taxa commonly considered as pests, such as cockroaches (Blattodea: Ectobiidae) and stink bugs (Hemiptera: Pentatomidae). Crickets and moths, both herbivorous insects that oftentimes forage on crops, were detected in a large proportion of gut samples. Moreover, the proportion of herbivorous arthropods consumed by nestlings was greater than the proportion of beneficial arthropods, such as predators and parasitoids (Fig 2). Bird nest boxes as well as bat houses can be added to agricultural areas, which if occupied, may enhance biological control of arthropod pests by vertebrates [60]. House wrens readily breed in nest boxes, therefore adding nest boxes to forest margins near agricultural fields and within urban natural areas may lead to fewer pests and a reduction in management strategies that can be harmful to the environment, such as chemical pesticides. Paired with molecular gut content analysis, a non-invasive technique, allows for monitoring the diets of vertebrate predators as well as the diversity of arthropod prey near boxes.

In addition to diet, other factors, such as noise and light pollution, may have direct or indirect effects on the behavior of adults or nestlings, which could influence nestling fitness. Low frequency anthropogenic noise masked begging calls of nestling tree swallows (*Tachycineta bicolor*), which led to missed detections and less frequent provisioning by adults [61]. On average, the urban study population of house wrens breed on louder territories [41], and therefore noise may affect detection of nestling begging calls in house wrens, either via signal masking or distraction. Increased noise levels may also lead parents to increase vigilance for predators near active nests, which may result in less time spend foraging [62]. Similarly, light pollution may increase nestling stress levels [63], which could lead to poor nestling health. However, artificial light at night may increase the amount of time during which adults can search for food and provision offspring. Northern mockingbirds (*Mimus polyglottos*) increased provisioning after dusk when exposed to artificial light at night [64]. In urban areas, light at night may make arthropods more available to adults, as birds may exploit arthropods that are attracted to artificial light [65]. Therefore, simultaneously considering multiple aspects of an urban environment is likely important to fully understand the effects of human-driven habitat change on animal populations.

Predation may also play a role in clutch sizes and nestling condition between urban and rural habitats if perceived risk of predation influences the reproductive strategy of adults [66]. For example, in an Arizona population of house wrens, when predators were removed from breeding grounds, clutch sizes declined but nestling body size increased compared to a control population [66]. In contrast, a Massachusetts population of house wrens exposed to predator playbacks at nests had both smaller clutch sizes and smaller nestlings [67]. In this population, nestling body condition also declined with increasing urbanization, suggesting that both urban habitats and a high risk of predation may interact to influence clutch size and nestling condition [67]. Despite a relatively large number of clutches (n = 42) that were lost during incubation, either due to predators or from destruction by other house wrens, nest failure post-hatch was rare (3.3%) in our study population. Thus, predation risk for nestlings was likely low for both urban and rural populations and other factors, such as provisioning rate of adults and quantity of prey delivered may explain better explain differences between body condition in our population.

Using a DNA metabarcoding approach, we complement measures of house wren nestling success and body condition with estimates diet composition and diversity. Overall, nestlings appear to be provisioned with a mixed diet composed of a diversity of arthropods, and that Orthoptera and Lepidoptera are the most commonly consumed orders of insects provided to nestlings. Nestling diets changed over the breeding season, becoming less diverse. Similarly, clutch sizes and early nestling survival also decreased seasonally. Whether these patterns are linked to local availability of prey during times of the year, preference for prey items, or quality of prey among sites is a question for future research.

## Supporting information

S1 Table Comparison of main effects and a model that included an interaction between habitat (urban or rural) and date using AICc and ΔAICc. (PDF)

S2 Table Frequency of taxa with greater than 10 reads per sample and total reads of prey items detected in House wren nestling gut samples by DNA metabarcoding. (PDF)

S1 Fig Map image of study sites in Kalamazoo, MI, USA. Sites included one rural location (Chipman Preserve, Southwest Michigan Land Conservancy) that was surrounded by agricultural fields, low density residential housing, and a commercial greenhouse (west of site). Nest boxes located at the urban natural areas were placed at Asylum Lake Preserve and the Western Michigan University Parkview Campus. Maps were generated in ArcMap 10.5 (Esri, Redlands, California, USA).
(TIFF)

S2 Fig Iterative rarefaction of sequencing depth. We explored a range of values for sequencing depth to standardize across samples prior to estimation of diversity indices, graphical analysis, or model fitting. Our goal was to retain as many samples as possible, while providing sufficient depth to capture all possible taxa or richness in samples. The analysis indicated, that for this particular dataset, a sequencing depth as low as 20 reads in a sample may be sufficient to estimate species richness. However, this seemed low, given that we set our threshold for positive tests at 10 reads per taxa (i.e. counts of presence). Therefore, we elected to use 50 reads as our threshold, which appeared to retain most samples and included full richness of taxa in fecal samples. The cost of increased sequencing depth is reduction in the number of samples meeting the depth criteria (50 reads = 183 samples, 100 reads = 177 samples, 200 reads = 165 samples, 400 reads = 151 samples).
(TIFF)

S3 Fig Comparison of diet metrics. Rarified relative read abundance (RRRA) and taxa occurrence (presence or absence) of the 2 most abundant prey items, Lepidoptera and Orthoptera, show similar patterns of detection according to date in house wren nestling diets.
(TIFF)

## References

[1] Hallmann CA, Sorg M, Jongejans E, Siepel H, Hofland N, Schwan H, et al. More than 75 percent decline over 27 years in total flying insect biomass in protected areas. PloS one. 2017;12(10):e0185809. doi: 10.1371/journal.pone.0185809 29045418 PMC5646769

[2] Rosenberg KV, Dokter AM, Blancher PJ, Sauer JR, Smith AC, Smith PA, et al. Decline of the North American avifauna. Science. 2019;366(6461):120–4. doi: 10.1126/science.aaw1313 31604313

[3] Wagner DL. Insect declines in the Anthropocene. Annual review of entomology. 2020;65:457–80. doi: 10.1146/annurev-ento-011019-025151 31610138

[4] McKinney ML. Effects of urbanization on species richness: a review of plants and animals. Urban ecosystems. 2008;11(2):161–76.

[5] Chamberlain DE, Cannon AR, Toms M, Leech DI, Hatchwell B, Gaston K. Avian productivity in urban landscapes: a review and meta-analysis. Ibis. 2009;151(1):1–18.

[6] Vaugoyeau M, Adriaensen F, Artemyev A, Bańbura J, Barba E, Biard C, et al. Interspecific variation in the relationship between clutch size, laying date and intensity of urbanization in four species of hole-nesting birds. Ecology and evolution. 2016;6(16):5907–20. doi: 10.1002/ece3.2335 27547364 PMC4983601

[7] Sol D, González-Lagos C, Moreira D, Maspons J, Lapiedra O. Urbanisation tolerance and the loss of avian diversity. Ecol Lett. 2014;17(8):942–50. doi: 10.1111/ele.12297 24835452

[8] Aronson MF, Lepczyk CA, Evans KL, Goddard MA, Lerman SB, MacIvor JS, et al. Biodiversity in the city: key challenges for urban green space management. Frontiers in Ecology and the Environment. 2017;15(4):189–96.

[9] Moretto L, Francis CM. What factors limit bat abundance and diversity in temperate, North American urban environments? Journal of Urban Ecology. 2017;3(1):jux016.

[10] Ouyang JQ, Isaksson C, Schmidt C, Hutton P, Bonier F, Dominoni D. A new framework for urban ecology: an integration of proximate and ultimate responses to anthropogenic change. Integrative and comparative biology. 2018;58(5):915–28. doi: 10.1093/icb/icy110 30376106 PMC6204990

[11] Shochat E, Warren PS, Faeth SH, McIntyre NE, Hope D. From patterns to emerging processes in mechanistic urban ecology. Trends in ecology & evolution. 2006;21(4):186–91. doi: 10.1016/j.tree.2005.11.019 16701084

[12] Buxton VL, Santymire RM, Benson TJ. Mixed effects of urbanization on density, nest survival, and nestling corticosterone of a generalist passerine. Ecosphere. 2018;9(12):e02517.

[13] Injaian AS, Taff CC, Patricelli GL. Experimental anthropogenic noise impacts avian parental behaviour, nestling growth and nestling oxidative stress. Animal Behaviour. 2018;136:31–9.

[14] Townsend AK, Staab HA, Barker CM. Urbanization and elevated cholesterol in American Crows. The Condor. 2019;121(3):duz040.

[15] Injaian AS, Poon LY, Patricelli GL. Effects of experimental anthropogenic noise on avian settlement patterns and reproductive success. Behavioral Ecology. 2018;29(5):1181–9.

[16] Seress G, Sándor K, Evans KL, Liker A. Food availability limits avian reproduction in the city: An experimental study on great tits *Parus major*. Journal of Animal Ecology. 2020;89(7):1570–80. 10.1111/1365-2656.13211 32419138

[17] Raubenheimer D, Simpson SJ, Mayntz D. Nutrition, ecology and nutritional ecology: toward an integrated framework. Funct Ecol. 2009;23(1):4–16.

[18] Razeng E, Watson DM. Nutritional composition of the preferred prey of insectivorous birds: popularity reflects quality. Journal of Avian Biology. 2015;46(1):89–96.

[19] Seress G, Bókony V, Pipoly I, Szép T, Nagy K, Liker A. Urbanization, nestling growth and reproductive success in a moderately declining house sparrow population. Journal of Avian Biology. 2012;43(5):403–14.

[20] Seress G, Hammer T, Bókony V, Vincze E, Preiszner B, Pipoly I, et al. Impact of urbanization on abundance and phenology of caterpillars and consequences for breeding in an insectivorous bird. Ecological Applications. 2018;28(5):1143–56. doi: 10.1002/eap.1730 29679462

[21] Shutt JD, Nicholls JA, Trivedi UH, Burgess MD, Stone GN, Hadfield JD, et al. Gradients in richness and turnover of a forest passerine’s diet prior to breeding: A mixed model approach applied to faecal metabarcoding data. Molecular ecology. 2020;29(6):1199–213. doi: 10.1111/mec.15394 32100904

[22] Mellott RS, Woods PE. An improved ligature technique for dietary sampling in nestling birds. Journal of Field Ornithology. 1993:205–10.

[23] Miller CK, McEwen LC. Diet of Nesting Savannah Sparrows in Interior Alaska. Journal of Field Ornithology. 1995:152–8.

[24] Valera F, GutiÉrrez JE, Barrios R. Effectiveness, biases and mortality in the use of apomorphine for determining the diet of granivorous passerines. The Condor. 1997;99(3):765–72.

[25] Serrano-Davies E, Sanz JJ. Habitat structure modulates nestling diet composition and fitness of Blue Tits *Cyanistes caeruleus* in the Mediterranean region. Bird Study. 2017;64(3):295–305.

[26] Wilkin TA, King LE, Sheldon BC. Habitat quality, nestling diet, and provisioning behaviour in great tits *Parus major*. Journal of Avian Biology. 2009;40(2):135–45.

[27] Clare EL, Fraser EE, Braid HE, Fenton MB, Hebert PD. Species on the menu of a generalist predator, the eastern red bat (Lasiurus borealis): using a molecular approach to detect arthropod prey. Molecular ecology. 2009;18(11):2532–42. doi: 10.1111/j.1365-294X.2009.04184.x 19457192

[28] Pompanon F, Deagle BE, Symondson WO, Brown DS, Jarman SN, Taberlet P. Who is eating what: diet assessment using next generation sequencing. Molecular ecology. 2012;21(8):1931–50. doi: 10.1111/j.1365-294X.2011.05403.x 22171763

[29] Deagle BE, Thomas AC, McInnes JC, Clarke LJ, Vesterinen EJ, Clare EL, et al. Counting with DNA in metabarcoding studies: How should we convert sequence reads to dietary data? Molecular ecology. 2019;28(2):391–406. doi: 10.1111/mec.14734 29858539 PMC6905394

[30] Bowers EK, Nietz D, Thompson CF, Sakaluk SK. Parental provisioning in house wrens: effects of varying brood size and consequences for offspring. Behavioral Ecology. 2014;25(6):1485–93.

[31] Dunn EH. The relationship between brood size and age of effective homeothermy in nestling House Wrens. The Wilson Bulletin. 1976;88(3):478–82.

[32] Styrsky JD, Eckerle KP, Thompson CF. Fitness–related consequences of egg mass in nestling house wrens. Proceedings of the Royal Society of London Series B: Biological Sciences. 1999;266(1425):1253–8.

[33] Young BE. Geographic and seasonal patterns of clutch-size variation in house wrens. The Auk. 1994;111(3):545–55.

[34] Burger C, Belskii E, Eeva T, Laaksonen T, Mägi M, Mänd R, et al. Climate change, breeding date and nestling diet: how temperature differentially affects seasonal changes in pied flycatcher diet depending on habitat variation. Journal of Animal Ecology. 2012;81(4):926–36. doi: 10.1111/j.1365-2656.2012.01968.x 22356622

[35] Hajdasz AC, Otter KA, Baldwin LK, Reudink MW. Caterpillar phenology predicts differences in timing of mountain chickadee breeding in urban and rural habitats. Urban Ecosystems. 2019;22(6):1113–22.

[36] Pollock CJ, Capilla-Lasheras P, McGill RA, Helm B, Dominoni DM. Integrated behavioural and stable isotope data reveal altered diet linked to low breeding success in urban-dwelling blue tits (Cyanistes caeruleus). Scientific reports. 2017;7(1):1–14. doi: 10.1038/s41598-016-0028-x 28694437 PMC5503996

[37] Greenewalt CH, Jones FM. Photographic studies of the feeding of nestling house wrens. Proceedings of the American Philosophical Society. 1955;99(4):200–4.

[38] Guinan DM, Sealy SG. Diet of house wrens (Troglodytes aedon) and the abundance of the invertebrate prey in the dune-ridge forest, Delta Marsh, Manitoba. Canadian Journal of Zoology. 1987;65(7):1587–96.

[39] Daan S, Dijkstra C, Drent R, Meijer T, editors. Food supply and the annual timing of avian reproduction. Proceedings of the International Ornithological Congress; 1988: University of Ottawa Press Ottawa.

[40] Dodson JC, Moy NJ, Bulluck LP. Prothonotary Warbler nestling growth and condition in response to variation in aquatic and terrestrial prey availability. Ecology and Evolution. 2016;6(20):7462–74. doi: 10.1002/ece3.2400 28725413 PMC5513273

[41] Grabarczyk EE, Araya-Salas M, Vonhof MJ, Gill SA. Anthropogenic noise affects female, not male house wren response to change in signaling network. Ethology. 2020;126(11):1069–78.

[42] Stuart CJ, Grabarczyk EE, Vonhof MJ, Gill SA. Social factors, not anthropogenic noise or artifical light, influence onset of dawn singing in a common songbird. Auk. 2019:1–10. doi: 10.1093/auk/ukz045

[43] Schulte-Hostedde AI, Zinner B, Millar JS, Hickling GJ. Restitution of mass–size residuals: validating body condition indices. Ecology. 2005;86(1):155–63.

[44] Hermans SM, Buckley HL, Lear G. Optimal extraction methods for the simultaneous analysis of DNA from diverse organisms and sample types. Molecular ecology resources. 2018;18(3):557–69. doi: 10.1111/1755-0998.12762 29394525

[45] Kitson JJ, Hahn C, Sands RJ, Straw NA, Evans DM, Lunt DH. Detecting host–parasitoid interactions in an invasive Lepidopteran using nested tagging DNA metabarcoding. Molecular ecology. 2019;28(2):471–83. doi: 10.1111/mec.14518 29485216

[46] Zeale MR, Butlin RK, Barker GL, Lees DC, Jones G. Taxon-specific PCR for DNA barcoding arthropod prey in bat faeces. Molecular ecology resources. 2011;11(2):236–44. doi: 10.1111/j.1755-0998.2010.02920.x 21429129

[47] Zhang J, Kobert K, Flouri T, Stamatakis A. PEAR: a fast and accurate Illumina Paired-End reAd mergeR. Bioinformatics. 2014;30(5):614–20. doi: 10.1093/bioinformatics/btt593 24142950 PMC3933873

[48] Schmidt JM, Whitehouse TS, Green K, Krehenwinkel H, Schmidt-Jeffris R, Sial AA. Local and landscape-scale heterogeneity shape spotted wing drosophila (Drosophila suzukii) activity and natural enemy abundance: Implications for trophic interactions. Agriculture, Ecosystems & Environment. 2019;272:86–94.

[49] Gordon A, Hannon G. Fastx-toolkit. FASTQ/A short-reads preprocessing tools (unpublished) http://hannonlab cshl edu/fastx_toolkit. 2010;5.

[50] Edgar RC. Search and clustering orders of magnitude faster than BLAST. Bioinformatics. 2010;26(19):2460–1. doi: 10.1093/bioinformatics/btq461 20709691

[51] Edgar RC. UPARSE: highly accurate OTU sequences from microbial amplicon reads. Nature methods. 2013;10(10):996–8. doi: 10.1038/nmeth.2604 23955772

[52] Tamura K, Dudley J, Nei M, Kumar S. MEGA4: molecular evolutionary genetics analysis (MEGA) software version 4.0. Molecular biology and evolution. 2007;24(8):1596–9. doi: 10.1093/molbev/msm092 17488738

[53] McClenaghan B, Nol E, Kerr KC. DNA metabarcoding reveals the broad and flexible diet of a declining aerial insectivore. The Auk: Ornithological Advances. 2019;136(1):uky003.

[54] Stolz C. The nestling diet of Svalbard snow buntings identified by DNA metabarcoding: UiT Norges arktiske universitet; 2019.

[55] Sullins DS, Haukos DA, Craine JM, Lautenbach JM, Robinson SG, Lautenbach JD, et al. Identifying the diet of a declining prairie grouse using DNA metabarcoding. The Auk: Ornithological Advances. 2018;135(3):583–608.

[56] Baldan D, Ouyang JQ. Urban resources limit pair coordination over offspring provisioning. Scientific reports. 2020;10(1):1–11. doi: 10.1038/s41598-019-56847-4 32985594 PMC7522258

[57] Narango DL, Tallamy DW, Marra PP. Nonnative plants reduce population growth of an insectivorous bird. Proceedings of the National Academy of Sciences. 2018;115(45):11549–54. doi: 10.1073/pnas.1809259115 30348792 PMC6233133

[58] Jarrett C, Powell LL, McDevitt H, Helm B, Welch AJ. Bitter fruits of hard labour: Diet metabarcoding and telemetry reveal that urban songbirds travel further for lower-quality food. Oecologia. 2020;193(2):377–88. doi: 10.1007/s00442-020-04678-w 32533359 PMC7320956

[59] Garcia K, Olimpi EM, Karp DS, Gonthier DJ. The Good, the Bad, and the Risky: Can Birds Be Incorporated as Biological Control Agents into Integrated Pest Management Programs? Journal of Integrated Pest Management. 2020;11(1):11.

[60] Lindell C, Eaton RA, Howard PH, Roels SM, Shave M. Enhancing agricultural landscapes to increase crop pest reduction by vertebrates. Agriculture, Ecosystems & Environment. 2018;257:1–11.

[61] Leonard ML, Horn AG. Ambient noise increases missed detections in nestling birds. Biology letters. 2012;8(4):530–2. doi: 10.1098/rsbl.2012.0032 22357939 PMC3391455

[62] Klett-Mingo JI, Pavón I, Gil D. Great tits, *Parus major*, increase vigilance time and reduce feeding effort during peaks of aircraft noise. Animal Behaviour. 2016;115:29–34.

[63] Raap T, Casasole G, Costantini D, AbdElgawad H, Asard H, Pinxten R, et al. Artificial light at night affects body mass but not oxidative status in free-living nestling songbirds: an experimental study. Scientific Reports. 2016;6(1):1–8. doi: 10.1038/s41598-016-0001-8 27759087 PMC5069498

[64] Stracey CM, Wynn B, Robinson SK. Light pollution allows the northern mockingbird (Mimus polyglottos) to feed nestlings after dark. The Wilson Journal of Ornithology. 2014;126(2):366–9.

[65] Lebbin DJ, Harvey MG, Lenz TC, Andersen MJ, Ellis JM. Nocturnal migrants foraging at night by artificial light. The Wilson Journal of Ornithology. 2007;119(3):506–8.

[66] Fontaine J, Martin T. Parent birds assess nest predation risk and adjust their reproductive strategies. Ecol Lett. 2006;9(4):428–34. doi: 10.1111/j.1461-0248.2006.00892.x 16623728

[67] Grade AM, Lerman SB, Warren PS. Perilous choices: landscapes of fear for adult birds reduces nestling condition across an urban gradient. Ecosphere. 2021;12(7):e03665.

