# Supplementary material for: Diet composition and diversity does not explain fewer, smaller urban nestlings
Source: PLoS One. 2022 Mar 1;17(3):e0264381. doi: 10.1371/journal.pone.0264381 (PMC8887731; doi:10.1371/journal.pone.0264381)
Supplement: S1 Table — (PDF) [file pone.0264381.s001.pdf]

S1 Table. Comparison of main effects and a model that included an interaction between habitat (urban or rural) and date using AICc and ∆AICc.

Parameter	Model	df	AICc	∆AICc
Clutch size	**Main**	**5**	**675.53**	
	Interaction	6	677.68	2.15
Survivorship	**Main**	**6**	**185.34**	
	Interaction	7	191.13	5.79
Body condition	**Main**	**7**	**22.92**	
	Interaction	8	30.9	7.98
