# Supplementary material for: Diet composition and diversity does not explain fewer, smaller urban nestlings
Source: PLoS One. 2022 Mar 1;17(3):e0264381. doi: 10.1371/journal.pone.0264381 (PMC8887731; doi:10.1371/journal.pone.0264381)
Supplement: S2 Table — (PDF) [file pone.0264381.s002.pdf]

S2 Table. Frequency of taxa with greater than 10 reads per sample and total reads of prey items detected in House wren nestling gut samples by DNA metabarcoding.

Taxonomic description				
Class/Subclass	Order	Family	Frequency of taxa with >10 reads per sample	Total reads
Insecta	Blattodea	Ectobiidae	3	13,848
	Coleoptera	Coccinellidae	0	226
		Curculionidae	1	40
		Elateridae	4	1,439
		Lampyridae	0	49
		Melandryidae	0	64
		Mycetophagidae	0	219
		Tenebrionidae	1	12,814
		**Coleoptera total**	**10**	**17,353**
	Dermaptera	Forficulidae	2	2,955
	Diptera	Asilidae	1	179
		Calliphoridae	2	238
		Cecidomyiidae	3	151
		Chloropidae	2	4,357
		Culicidae	0	23
		Drosophilidae	0	11
		Limoniidae	1	5,826
		Muscidae	1	189
		Phoridae	1	261
		Pipunculidae	0	32
		Rhagionidae	5	4,303
		Tachinidae	4	6,983
		Therevidae	0	167
		Tipulidae	3	39,868
		**Diptera total**	**29**	**90,879**
	Hemiptera	Alydidae	0	69
		Aphididae	0	126
		Aphrophoridae	8	35,832
		Cicadellidae	14	34,546
		Clastopteridae	2	585

		Miridae	0	28
		Pentatomidae	5	7,303
		**Hemiptera total**	**33**	**79,527**
	Hymenoptera	Aphelinidae	0	15
		Braconidae	3	1,624
		Diapriidae	1	28
		Ichneumonidae	10	23,630
		Mutillidae	1	61
		Perilampidae	2	9,912
		Tenthredinidae	2	598
		**Hymentoptera total**	**93**	**32,8473**
	Lepidoptera	Depressariidae	2	507
		Erebidae	29	67,210
		Geometridae	12	17,854
		Hesperiidae	2	1,515
		Limacodidae	3	468
		Lycaenidae	5	12,123
		Noctuidae	71	181,224
		Nolidae	1	25
		Notodontidae	7	39,898
		Nymphalidae	1	8,886
		Tortricidae	4	4,873
		**Lepidoptera total**	**149**	**353,599**
	Mantodea	Mantidae	8	14,989
	Mecoptera		2	237
	Neuroptera	Chrysopidae	2	4,381
		Hemerobiidae	2	5,759
		Mantispidae	2	2,778
		**Neuroptera total**	**6**	**12,918**
	Orthoptera	Acrididae	12	3,832
		Gryllidae	96	279,729
		Rhaphidophoridae	0	65
		Tetrigidae	2	3,112
		Tettigoniidae	11	34,234
		Trigonidiidae	15	25,600
		**Orthoptera total**	**248**	**1,116,880**
	Psocodea	Amphipsocidae	1	10,585
	Psocodea	Peripsocidae	0	60
Collembola	Symphypleona		0	45
Arachnida	Araneae	Agelenidae	4	1,677

		Anyphaenidae	0	107
		Araneidae	1	72
		Corinnidae	2	762
		Lycosidae	17	39,662
		Oxyopidae	1	767
		Philodromidae	11	33,785
		Pisauridae	12	22,907
		Salticidae	14	23,249
		Theridiidae	2	1,393
		Thomisidae	13	11,596
		**Araneae total**	**91**	**171,970**
Arachnida/Acari	Sarcoptiformes	Oribatellidae	1	29
	Sarcoptiformes	Scheloribatidae	0	18
	Trombidiformes	Eupodidae	2	804
Diplopoda	Polydesmida	Paradoxosomatidae	38	143,685
	Julida	Julidae	2	269
Branchiopoda	Diplostraca	Daphniidae	1	7,643
Malacostraca	Isopoda	Trachelipodidae	2	3,777
