# Supplementary figures and images for: Diet composition and diversity does not explain fewer, smaller urban nestlings
Source: PLoS One. 2022 Mar 1;17(3):e0264381. doi: 10.1371/journal.pone.0264381 (PMC8887731; doi:10.1371/journal.pone.0264381)

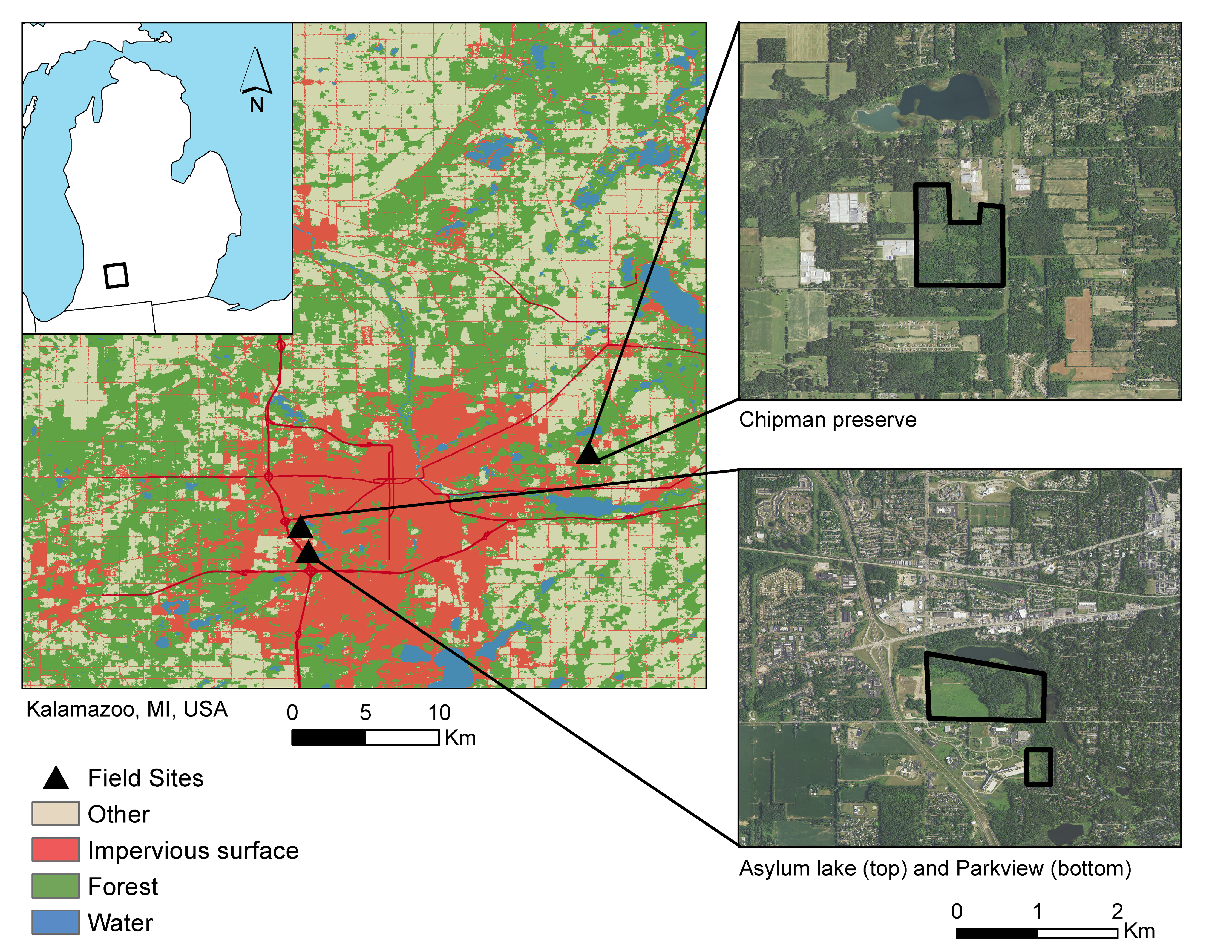

Supplement: S1 Fig — Sites included one rural location (Chipman Preserve, Southwest Michigan Land Conservancy) that was surrounded by agricultural fields, low density residential housing, and a commercial greenhouse (west of site). Nest boxes located at the urban natural areas were placed at Asylum Lake Preserve and the Western Michigan University Parkview Campus. Maps were generated in ArcMap 10.5 (Esri, Redlands, California, USA). (TIFF) [file pone.0264381.s003.tiff]

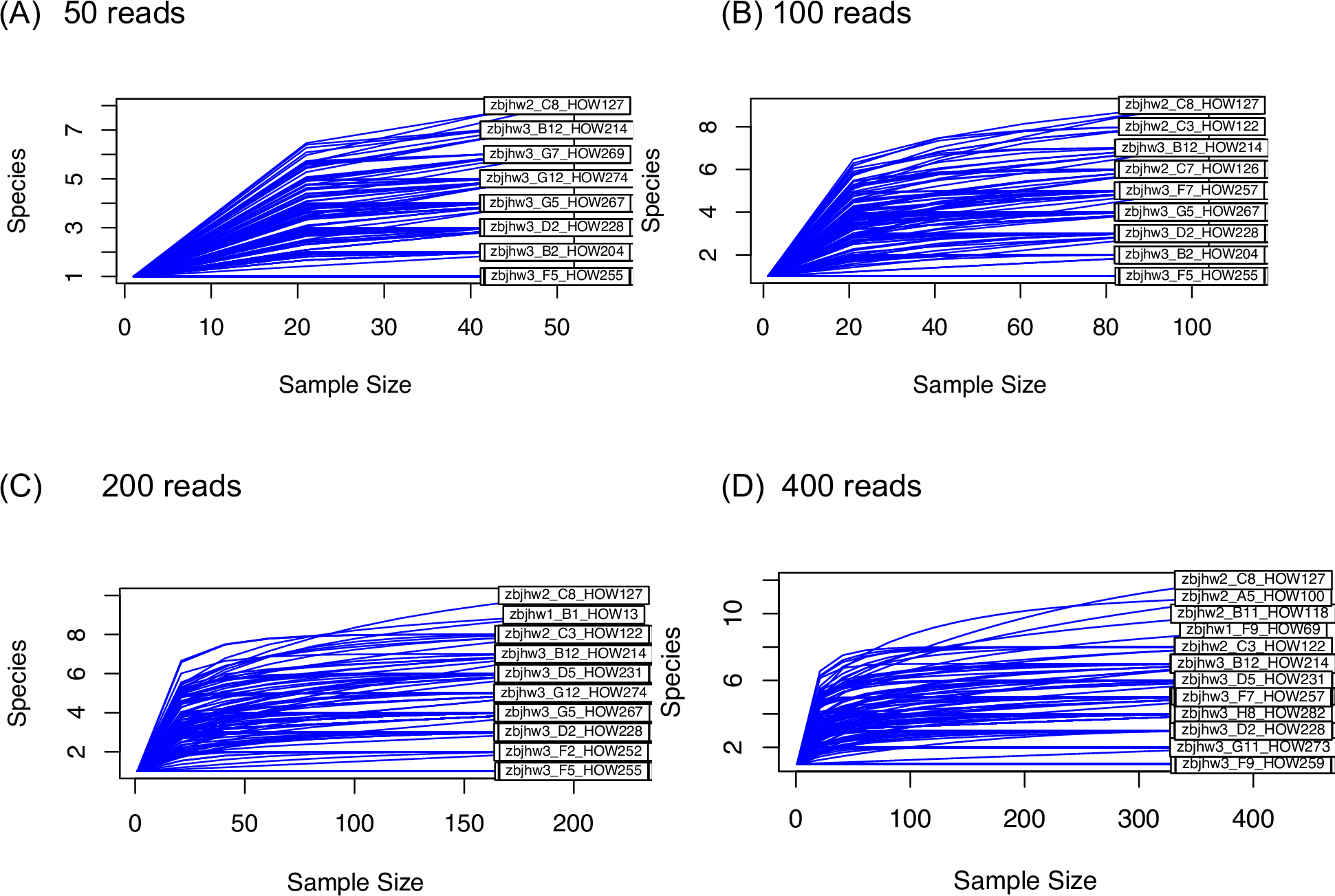

Supplement: S2 Fig — We explored a range of values for sequencing depth to standardize across samples prior to estimation of diversity indices, graphical analysis, or model fitting. Our goal was to retain as many samples as possible, while providing sufficient depth to capture all possible taxa or richness in samples. The analysis indicated, that for this particular dataset, a sequencing depth as low as 20 reads in a sample may be sufficient to estimate species richness. However, this seemed low, given that we set our threshold for positive tests at 10 reads per taxa (i.e. counts of presence). Therefore, we elected to use 50 reads as our threshold, which appeared to retain most samples and included full richness of taxa in fecal samples. The cost of increased sequencing depth is reduction in the number of samples meeting the depth criteria (50 reads = 183 samples, 100 reads = 177 samples, 200 reads = 165 samples, 400 reads = 151 samples). (TIFF) [file pone.0264381.s004.tiff]

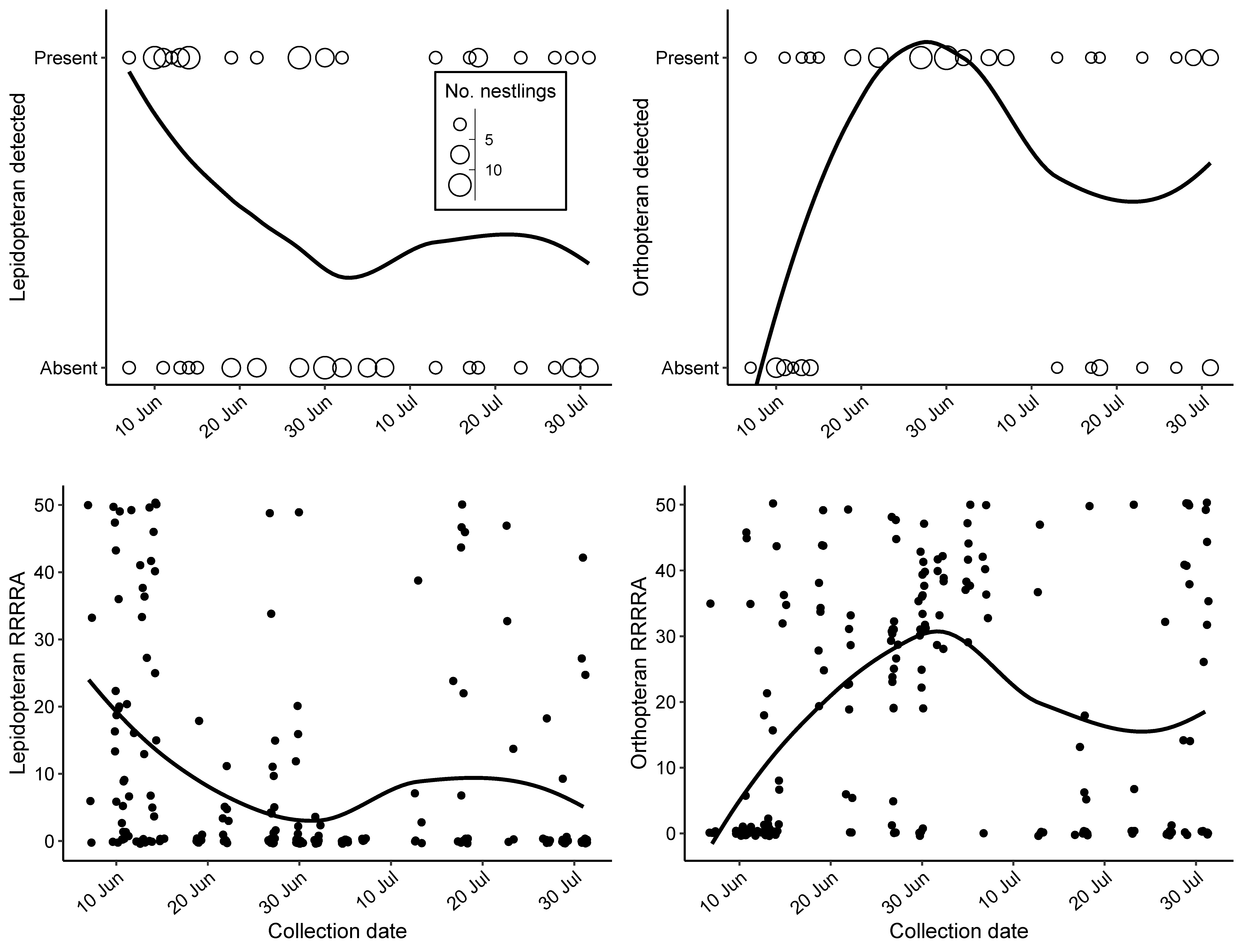

Supplement: S3 Fig — Rarified relative read abundance (RRRA) and taxa occurrence (presence or absence) of the 2 most abundant prey items, Lepidoptera and Orthoptera, show similar patterns of detection according to date in house wren nestling diets. (TIFF) [file pone.0264381.s005.tiff]
